# Lamina I NK1 expressing projection neurones are functional in early postnatal rats and contribute to the setting up of adult mechanical sensory thresholds

**DOI:** 10.1186/1744-8069-8-35

**Published:** 2012-04-27

**Authors:** Sharon HW Man, Sandrine M Géranton, Stephen P Hunt

**Affiliations:** 1 Department of Cell and Developmental Biology, University College London, London, WC1E 6BT, UK

**Keywords:** Lamina 1, Projection neurones, Parabrachial nucleus, c-fos, NK1, Formalin, Postnatal rat, Substance-P saporin, Mechanical sensory thresholds

## Abstract

**Background:**

A small proportion of lamina I neurons of the spinal cord project upon the hindbrain and are thought to engage descending pathways that modulate the behavioural response to peripheral injury. Early postnatal development of nociception in rats is associated with exaggerated and diffuse cutaneous reflexes with a gradual refinement of responses over the first postnatal weeks related to increased participation of inhibitory networks. This study examined the postnatal development of lamina I projection neurons from postnatal day 3 (P3) until P48.

**Results:**

At P3, a subset of lamina I neurons were found to express the neurokinin 1 (NK1) receptor. Using fluorogold retrograde tracing, we found that the NK1 positive neurons projected upon the parabrachial nucleus (PB) within the hindbrain. Using c-fos immunohistochemistry, we showed that lamina I and PB neurons in P3 rats responded to noxious stimulation of the periphery. Finally, ablation of lamina I neurons with substance-P saporin conjugates at P3 resulted in increased mechanical sensitivity from P45 onwards compared to control animals of the same age.

**Conclusions:**

These results suggest that the lamina I pathway is present and functional at least from P3 and required for establishing and fine-tuning mechanical sensitivity in adult rats.

## Introduction

In the adult rat, projections from a discrete population of lamina I neurons regulate the increase in mechanical and thermal sensitivity that develops after injury [1-3]. These superficial projection neurons express the neurokinin 1 (NK1) receptor (the preferred receptor for substance P) (SP) [1,4], receive inputs from peripheral nociceptors [5,6], support long-term potentiation following high threshold stimulation of incoming nociceptors [5,7,8] and project to the parabrachial nucleus (PB), periaqueductal gray (PAG) and various parts of the medulla and thalamus [9,10]. Some projection neurons may also send collaterals locally into deeper laminae of the dorsal horn [11]. Ablation of these lamina I neurons with saporin-substance P conjugates results in a failure to maintain the persistent mechanical hyperalgesia seen in both inflammatory and neuropathic pain models [2]. Previous research has implied that lamina I projections form the first stage of a spinal-brainstem-spinal loop that is necessary for the induction and maintenance of pain states [3,12]. NK1-expressing lamina I neurons indirectly activate descending pathways originating in the rostral ventromedial medulla (RVM) possibly through a PAG-RVM pathway. Indeed, lesions of the RVM or pharmacological inhibition of specific pathways originating in the RVM, such as mu-opiate receptor-expressing ON neurons or serotonin–expressing neurons, results in a loss of the increased pain sensitivity seen in experimental pain models [13,14]. It has also been demonstrated electrophysiologically that lesions of the ascending lamina I pathway or pharmacological inhibition of the descending serotonergic pathway at spinal level reduces spinal nociceptive transmission in mature rats [3,15,16].

Spinal processing of nociceptive stimuli begins before birth and matures postnatally [17]. Nociceptive networks are more excitable in young postnatal rats and exaggerated and diffuse cutaneous reflexes are seen in neonates. Cutaneous responses mature to adult levels in the weeks following birth, and this is accompanied by a gradual increase in descending inhibition from the RVM [18]. However, little is known about the functional development of the lamina I NK1-expressing pathway after birth. Previous studies have shown that SP is expressed by C-fibres before birth and stimulation of the dorsal root at high intensities can stimulate release of SP in neonatal rat spinal cord [19,20]. The NK1 receptor has been localized to superficial dorsal horn neurons at postnatal day fourteen (P14) [21] but not at earlier times in the rat dorsal horn. Here, we show that neonatal lamina I neurons express NK1 receptors and project to the parabrachial nucleus (PB) at least from P3. We also show that at this developmental stage noxious stimulation of the periphery results in activation of NK1-positive projection neurons and post-synaptic target neurons within the PB. Finally, we show that early ablation of lamina I neurons at P3 affects the setting of adult mechanical sensitivity.

## Materials and methods

### Animal preparation

All procedures complied with the United Kingdom Animals (Scientific Procedures) Act 1986. Experiments were performed on male Sprague–Dawley postnatal day 3 (P3), P10, P20 and adult rats (P48 onwards; 200–250 g) from the colony at University College London. Animals were kept in their home cages at 21°C and 55% relative humidity with a 12 hour light/dark cycle (lights on at 08:00 h) and had unlimited access to food and water. All efforts were made to minimize animal suffering and to reduce the number of animals used.

### Antibodies and drugs

Anti-NK1 receptor antibody was a gift from S. Vigna; anti-c-fos was from Calbiochem (Darmstadt, Germany); anti-Fluorogold (anti-FG) from Fluorochrome (Englewood, NJ, USA); anti-NeuN, anti-5-HT and anti-CGRP from Chemicon (CA, US); anti-GFAP from DakoCytomation (Denmark); anti-PKC gamma from Santa Cruz (CA, USA); and anti-isolectin B4 from Vector Laboratories (CA, USA). Formalin was purchased from BDH; Fluorogold (FG) was from Fluorochrome (Englewood, NJ, USA). Substance P-Saporin (SP-SAP) and Blank- Saporin (blank-SAP) were from Advanced Targeting Systems (San Diego, California).

### Formalin stimulation

2.5% formalin diluted in 0.9% normal saline was used to induce expression of c-fos in the spinal cord. To take into account the postnatal increase in the size of hindpaw, the volumes of formalin solution injected in P3, P10, P20 and adult rats were 10 μl, 20 μl, 40 μl and 100 μl respectively. These injection volumes were optimized in pilot studies to cover a similar proportion of the animals’ hindpaw across all ages. Injection was delivered with a 0.5 ml insulin syringe with a 28 G needle. Prior to injection of formalin, animals were manually restrained, without anaesthesia, in plantar flexion. Subcutaneous injection of formalin was performed on the plantar surface of the left hindpaw. To control for restraint-induced stress in formalin-injected animals, control animals were also briefly restrained in plantar flexion. Following injection of formalin or brief restraint, P3 and P10 rats were separated from their mothers and kept in a box lined with a thermal pad for two hours. P20 rats and adult rats were returned to their home cages and kept for two hours. Maternal separation of young rats was performed to reduce variability in basal c-fos expression induced by maternal grooming and feeding [22]. Expression of c-fos typically peaks at 2 hours following physiological stimulation (Hunt et al., 1987; Herdegen and Leah, 1998). Therefore, animals were perfused at 2 hours after injection of formalin

### Fluorogold injections

4% Fluorogold (FG) mixed in distilled water was prepared on the day of injection. A gas mixture of 4% halothane and 100% oxygen delivered at 2 L/min in a close chamber was used to induce anaesthesia in P3 rats and adult rats. The rats were placed in a Kopf stereotaxic frame and anaesthesia was maintained by the delivery of 1.5 – 2% halothane combined with 100% O2 (1 L/min) via a face mask. A small incision was made in the scalp to expose the skull and reveal bregma. Following craniotomy, animals received an injection of 4% FG into the lateral parabrachial nucleus (PB) on the right side (coordinates for P3: –5.2 mm anteroposterior, 1.3 mm mediolateral and −5.0 mm dorsoventral; coordinates for adult: -9.2 mm anteroposterior, 1.7 mm mediolateral and −6.4 mm dorsoventral) delivered by a 2.5 μl Hamilton syringe. These coordinates were obtained from pilot studies based on an atlas of the rat brain [23]. The injection volumes of FG in P3 and adult rats obtained from pilot studies were 50 nl and 300 nl respectively. Rats were allowed to recover from anaesthesia in an incubation chamber and then transferred back to their home cages until perfusion.

On the day of perfusion (1 day after FG injection in P3 rats and 3 days after FG injection in adult rats), rats were perfused in their naïve state or received a subcutaneous injection of formalin in the left hindpaw, and perfused two hours later. Following fixation and cryoprotection, the brain and the spinal cord were then sectioned on a freezing microtome. Sections were then mounted onto glass slides under a fluorescence microscope equipped with a wide band UV filter.

### Substance P-Saporin (SP-SAP) injections

P3 rats were briefly anaesthetized with a mixture of 4% halothane and 100% oxygen delivered at 2 L/min. A 5 μl Hamilton syringe (model 84851, Essex Scientific Laboratory Support) with a removable needle (gauge 32, 25 mm in length, point style 4) was used to deliver the drug intrathecally. Intrathecal injection was targeted at the level of the sixth lumbar vertebrae to minimize damage to the neonatal spinal cord. The needle was aimed at the midpoint of the vertebral column just above the pelvic girdle and carefully advanced until a slight decrease in resistance was felt in the path of the needle and a small flick of the tail or of the lower limb was observed. These signs indicated entry of the needle into the intrathecal space. 2 μl of SP-SAP or blank-SAP at 5 μM was then injected manually over one minute in P3 rats. At the end of the injection, the needle was slowly withdrawn. Rats were allowed to recover from anaesthesia in an incubation chamber and then transferred back to their home cages. Locomotive abilities, feeding behaviours and maternal-neonatal interaction were monitored every day in the first week and then once every 3 days until P48. Body weights of animals were also recorded postnatally to monitor growth. Mechanical threshold of the left hindpaw was measured prior to treatment at P3 and postnatally to assess cutaneous reflex of the lower limbs until P48. Animals were then terminally anaesthetized and perfused for histology.

### Behavioural assay

Von Frey filaments (Stoelting, IL, USA) were used to test mechanical threshold of the hindpaw of postnatal rats treated with SP-SAP and control postnatal rats. All measurements were performed blind to treatment allocation. Testing was an adapted ‘up-down’ method [24]. Prior to testing, animals were placed in clear perspex compartments situated above a metal wire mesh that allowed access to the plantar surfaces of the animals’ hindpaws. Animals were allowed to habituate to the testing environment for 15 min prior to testing. A series of up to 13 Von Frey filaments with logarithmically incremental stiffness was applied perpendicularly to the mid-plantar surface of the hindpaw until slightly bent. Each filament was applied 5 times with 2 to 3 seconds in between applications. A positive response was inferred from a reflex withdrawal of the stimulated hindpaw from a filament. Mechanical threshold was the lowest Von Frey filament that elicited more than 2 positive responses out of 5 applications.

### Immunohistochemistry

Rats were deeply anaesthetized with intraperitoneal pentobarbital and perfused transcardially with saline containing 5 000 I.U./ml heparin followed by 4% paraformaldehyde (PFA) in 0.1 M phosphate buffer (50 ml, 100 ml, 150 ml and 250 ml in P3, P10, P20 and adult rats respectively). Lumbar spinal cord was dissected out, post-fixed in the same PFA solution for 2 hours and transferred into a 30% sucrose solution in phosphate buffer containing 0.01% azide, for a minimum of 24 hours, at 4°C. Spinal cords were cut on a freezing microtome set at 40 μm. All antibody solutions contained 0.01% triton X-100 and 0.3% serum of the host species of a secondary antibody to block non-specific background staining. Sections were incubated with primary antibodies for 48 hours at 4°C. The primary antibodies used were anti-NK1 receptor (1:5000 for biotin protocol or 1:100,000 with tyramide signal amplification (TSA) protocol), anti-c-fos (1:5000 with biotin protocol and 1:100,000 with TSA protocol), anti-Fluorogold (1:50,000 for direct stain), anti-NeuN (1:1000 for direct stain), GFAP (1:1000 with biotin protocol), PKC gamma (1:2000 with biotin protocol), CGRP (1:2000 with biotin protocol), 5-HT (1:100, with biotin protocol) and isolectin B4 (IB4) (1:500, with biotin protocol). For the biotin protocol, appropriate secondary biotinylated antibodies were used (1:500, 2 h) followed by avidin-Cy3 (1:4000, 1 hour, Vector Labs) or avidin-FITC (1:2000, 2 hours, Vector Labs).

For the TSA protocol, following incubation in primary antibody, sections were first incubated with appropriate secondary biotinylated antibodies (1:400, 1.5 hour) followed by avidin biotin complex (ABC Elite; 1:250 Vectastain A plus 1:250 Vectastain B; Vector Laboratories) for 30 minutes followed by a signal amplification step with biotinylated tyramide solution (1:75 for 7 minutes, Perkin Elmer). Sections were then incubated with FITC avidin for a further 2 hours (1:600). Finally, sections were incubated with the second primary antibody overnight at room temperature, followed by incubation in appropriate Alexa Fluor (1:500, 2 h).

NK1-c-fos, FG-NK1 and FG-c-fos double immunostaining were obtained with the TSA protocol. NeuN-c-fos double immunostaining and single immunostaining for NK1, NeuN, GFAP, PKC gamma, CGRP and 5-HT were obtained with the biotin protocol.

All sections were coverslipped with Gel Mount aqueous mounting medium (Sigma) to preserve fluorescence and stored in the dark at 4°C. Controls for immunohistochemistry were carried out omitting the first or second primary antibodies. In some cases, Fluorogold staining was directly visualized with UV detection.

### Image acquisition and quantification of immunostaining

Imaging systems connected with Nikon Eclipse E800 microscope or Leica DMRBE confocal microscope equipped with SP2 confocal head were used to acquire images of spinal cord and brain sections immunostained with different antibodies. Images captured from the left dorsal horn of animals injected with formalin in the left hindpaw were used to quantify lamina I neurons immunostained for NK1, c-fos or FG in P3 and adult rats. Neurons with positive immunostaining for NK1, c-fos or FG along the whole length of lamina I were counted. Colocalization of NK1 with c-fos, NK1 with FG or FG with c-fos was assessed using single focal planes.

Images captured from the right lateral parabrachial nucleus (PB) and the left dorsal horn of animals injected with formalin in the left hindpaw were used to quantify parabrachial neurons and lamina I neurons immunostained for NeuN or c-fos in postnatal and adult rats. Colocalization of NeuN with c-fos was assessed using single focal planes. Using the drawing tool in Photoshop, a rectangular box (300 μm wide × 150 μm deep) was placed lateral to the superior cerebellar peduncle to quantify PB neurons immunostained for NeuN or c-fos. Likewise a rectangular box (250 μm wide × 50 μm deep) was placed on the medial part of lamina I to quantify lamina I neurons immunostained for NeuN or c-fos. Neuronal density was obtained by counting the number of neurons labelled with NeuN antibody within the rectangular boxes. A previous study showed that the superficial dorsal horn is approximately 200 μm, 250 μm and 300 μm deep from the dorsal white matter in P3, P10 and P21 rats respectively [25]. To account for change in neuronal density with age, we calculated percentages of NeuN + ve lamina I and PB neurons that co-localized with c-Fos in order to compare levels of activation of lamina I projection pathway between different postnatal ages.

### Statistical analysis

Independent *t*-test was applied to test for difference in mean number of FG-labelled lamina I projection neurons and mean percentage of FG-labelled lamina I projection neurons that expressed c-fos between P3 and adult rats. One-way ANOVA was used to test for effect of age on neuronal density in the PB and percentage of PB neurons that expressed c-fos. Multiple pairwise comparison with post-hoc Bonferroni test were carried out to test for difference between group means. Median test was used to test for effect of age on lamina I neuronal density as the data was non-parametric. Mann–Whitney test for non-parametric data was used to test for difference in lamina I neuronal density between age groups. One-way ANOVA was used to test for effect of age on the percentage of lamina I neurons that expressed c-fos.

Difference in mechanical threshold at different postnatal time points between postnatal rats treated with SP-SAP and control rats was assessed using repeated measure ANOVA and relevant post-hoc analysis. Since there was no significant difference in body weight or mechanical threshold between naïve rats and rats injected with blank-SAP across the whole study, data from these two groups were combined together into one control group. Statistical significance was set at a level of p < 0.05 in all statistical tests performed in this study.

## Results

### NK1-positive lamina I neurons express c-fos in response to noxious stimulation at P3 and project to the parabrachial area

**Figure 1:**
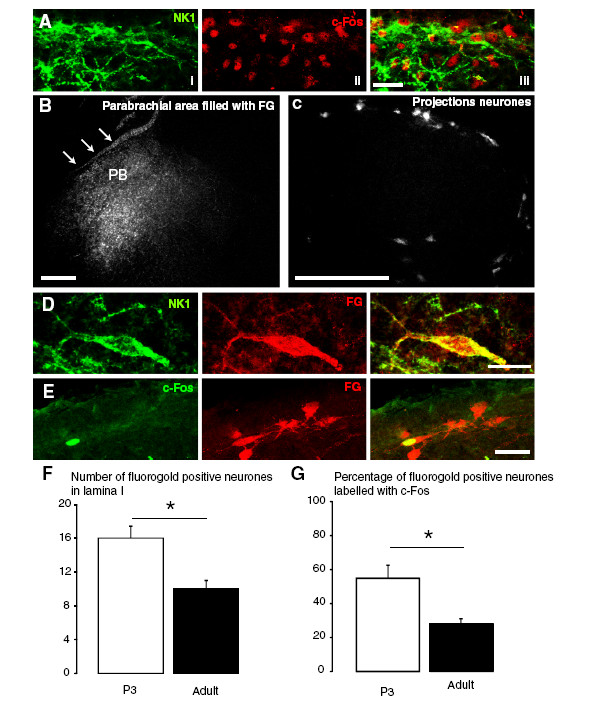
**NK1-positive lamina I neurons express c-fos in response to injection of formalin in the hindpaw and project to the lateral parabrachial nucleus (PBN) in postnatal day 3 (P3) rats. A,** Immunohistochemistry for NK1 (green) and c-fos (red) in the dorsal horn of a formalin-stimulated P3 rat. Colocalization of NK1 with c-fos can be seen. Scale bar, 40 μm. **B,** Fluorogold (FG) injection site in the lateral PBN. Arrows indicate the edge of the brain section. PB: parabrachial area. **C**, Retrograde labeling of lamina I-parabrachial neurons with FG in a P3 lumbar cord section at 24 hours post-injection. **B and C:** Scale bar, 200 μm. **D**, Immunohistochemistry for NK1 (green) and FG (red) in lamina I at 24 hours after injection of FG in the lateral PBN at P3. Co-localization of NK1 with FG is seen in yellow. Scale bar, 20 μm. **E,** Immunohistochemistry for c-fos (green) and FG (red) in lamina I of a formalin-stimulated P3 rat 1 day after parabrachial injection with FG. Co-localization of c-fos with FG can be seen in yellow. Scale bar, 40 μm. **F**, Number of lamina I neurons labeled with FG in P3 (N = 4) and adult rats (N = 3) injected with FG in the lateral PBN. **G,** Percentage of FG-labeled neurons in lamina I that co-localized with c-fos after injection of formalin in P3 (N = 4) and adult rats (N = 3). Data show group mean ± SEM. * *p* < 0.05.

In transverse sections of the spinal cord NK1-positive neurons were observed with dendrites extending in parallel to the dorsal margin of the dorsal horn in lamina I of P3 rats (Figure 1Ai). Double-immunostaining for c-fos and NK1 in the formalin-stimulated P3 spinal cord revealed that an average of 3 neurons (2.8 ± 0.2) per section co-expressed c-fos and NK1 in lamina I (Figure 1Aiii, N = 3, number of sections per animal = 3). These neurons represented 85.3% ± 8.1% of the NK1 cells. Injection of fluorogold (FG) in the lateral parabrachial nucleus (PB) in P3 (Figure 1B) resulted in retrograde labelling of lamina I-PB projection neurons (Figure 1C). Some of these neurons also expressed NK1 receptors (Figure 1D). The mean percentage of retrogradely labelled lamina I-PB neurons that expressed NK1 in P3 was 40.8 ± 3.8% (N = 3) c-fos expression was also observed in lamina I-PB projection neurons in P3 rats stimulated by formalin injection in the hindpaw (Figure 1E). We found that more lamina I-PB neurons were retrogradely labelled by FG in P3 rats compared to adult rats (16 ± 1 *vs* 10 ± 1 = 6 neurons per section, *p* < 0.05, N = 3–4) (Figure 1F). Formalin injection in the hindpaw stimulated c-fos expression in a higher proportion of lamina I-PB neurons in P3 rats compared to adult rats (54.8 ± 7.6% *vs* 28.0 ± 2.8% = 26.8%, *p* < 0.05, N = 3–4) (Figure 1G).

### The c-fos response of dorsal horn neurons does not change with postnatal age following peripheral noxious stimulation

**Figure 2:**
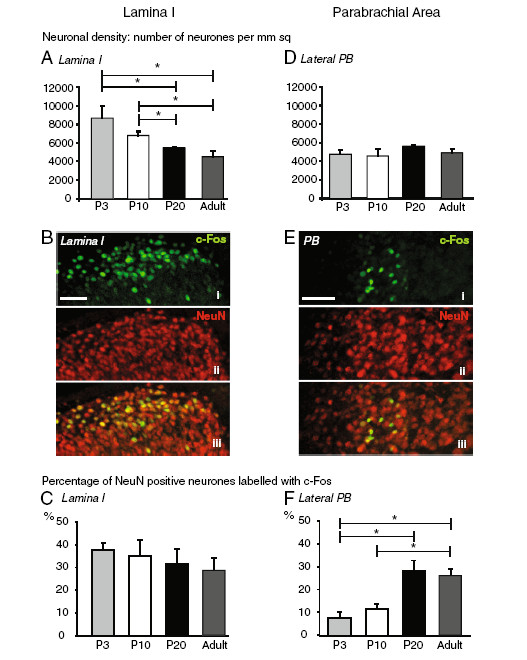
**c-fos expression stimulated by injection of formalin in the hindpaw increases with postnatal age in the lateral parabrachial nucleus (PB) but not in the superficial dorsal horn. A**, Neuronal density in lamina I of dorsal horn decreases with postnatal age. **B,** Immunohistochemistry for c-fos (green) and NeuN (red) in the dorsal horn of a P3 rat two hours after injection of formalin in the hindpaw. Co-localization of c-fos with NeuN in the same section of dorsal horn can be seen in yellow. Scale bar, 100 μm. **C,** Percentage of NeuN-positive lamina I neurons that expressed c-fos after injection of formalin in the hindpaw in P3 (N = 3), P10 (N = 5), P20 (N = 3) and adult (N = 5) rats. Injection of formalin in the neonatal rat hindpaw induced similar level of stimulation of lamina I neurons to that in the adult rat. **D,** Neuronal density in the lateral PB remained constant with postnatal age. **E,** Immunohistochemistry for c-fos (green) and NeuN (red) in the lateral PBN of a P3 rat at 2 h after injection of formalin in the hindpaw. Co-localization of c-fos with NeuN in the same section of the lateral PB can be seen in yellow. Scale bar, 200 μm. **F,** Percentage of NeuN-positive lateral PBN neurons that expressed c-fos after injection of formalin in the hindpaw in P3 (N = 3), P10 (N = 2), P20 (N = 3) and adult (N = 4) rats. Data show mean ± SEM. * *p* < 0.05.

Postnatal age had a significant effect on lamina I neuronal density (chi-square = 9.6(3), *p* < 0.05, Median test; Figure 2A). Lamina I neuronal density in P3 and P10 rats were greater than in P20 and adults rats (*p* < 0.05; Figure 2A). However, the neuronal density in lamina I did not differ between P3 and P10 rats, indicating that a significant decrease in lamina I neuronal density occurred between P10 and P20. Unilateral injection of formalin in the hindpaw stimulated c-fos expression in the ipsilateral dorsal horn in P3 rats (Figure 2Bi). However postnatal age had no significant effect on the percentage of lamina I neurons that expressed c-fos (F_3, 12_ = 0.39, *p* = 0.77, one way ANOVA; Figure 2C) (N = 3–5). The percentages of lamina I neurons that expressed c-fos in formalin-stimulated P3, P10, P20 and adult rats were 37.5 ± 3.0%, 35.0 ± 6.9%, 31.7 ± 6.3% and 28.9 ± 5.1% respectively. This implies that the level of stimulation of lamina I neurons by injection of formalin in the hindpaw of neonatal rats was similar to that in adult rats. No c-fos expression was observed in lamina I of unstimulated control animals at any of the ages studied.

### The c-fos response of parabrachial neurons increased with postnatal age following peripheral noxious stimulation

Postnatal age had no significant effect on PB neuronal density (Figure 2D). Unilateral injection of formalin in the hindpaw stimulated c-fos expression in PB (Figure 2Ei) and one-way ANOVA analysis of c-fos positive cells in the contralateral PB of formalin-stimulated rats showed that age had significant effect on percentage of PB neurons that expressed c-fos (F_3,8_ = 9.35, *p* < 0.01; Figure 2F). The mean percentages of PB neurons that expressed c-fos in formalin-stimulated P3, P10, P20 and adult rats were 7.7 ± 2.5%, 11.6 ± 3.4%, 27.7 ± 4.6% and 26 ± 2.5% respectively (N = 3–4). *Post hoc* analysis showed that the percentages of PB neurons that expressed c-fos at P20 and in adult rats were greater than that at P3 (*p* < 0.05). Likewise the percentage of PB neurons that expressed c-fos in adult rats was also greater than that at P10 and P3 (*p* < 0.05). No c-fos expression was observed in the PB of unstimulated control animals at any of the ages studied.

### Neonatal SP-SAP treatment at P3 resulted in selective depletion of NK1-positive lamina I/III neurons at P48

**Figure 3:**
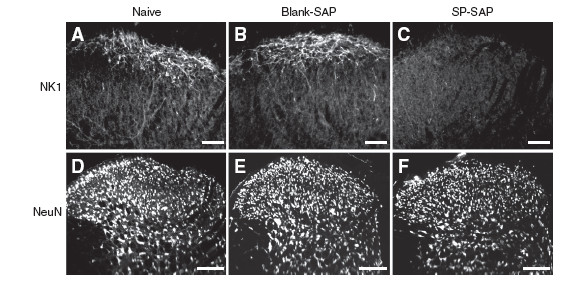
**Neonatal substance P-saporin (SP-SAP) treatment selectively depletes NK1 + ve laminae I/III neurons at P48. A, B, C,** Immunohistochemistry for NK1 in naïve rats (Figure 3A), rats treated with blank-saporin (blank-SAP) (Figure 3B) and rats treated with SP-SAP (Figure 3C) respectively. Scale bar, 50 μm. **D, E, F,** Immunohistochemistry for NeuN in naïve rats (Figure 3D), rats treated with blank-SAP (Figure 3E) and rats treated with SP-SAP (Figure 3F) respectively. Scale bar, 100 μm.

**Figure 4:**
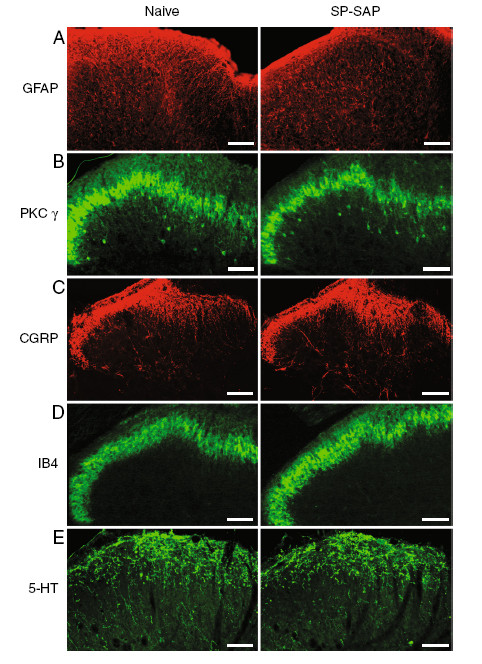
**Neonatal SP-SAP treatment has no effect on expression of GFAP, PKCγ, CGRP, IB4 and 5-HT in the superficial dorsal horn at P48. **Immunohistochemistry for GFAP (Figure 4A), PKCγ (Figure 4B), CGRP (Figure 4C), IB4 (Figure 4D), 5-HT (Figure 4E) in dorsal horn of naïve rats and rats treated with SP-SAP at P48. Scale bar, 50 μm.

There was no difference in expression of NK1-positive dorsal horn neurons between naïve P48 rats and P48 rats treated with blank-SAP at P3 (Figure 3F and 3B). In these animals, NK1-expressing dendrites and occasionally NK1-expressing cell bodies were observed in lamina I/III. However, in P48 rats treated with SP-SAP at P3, there was a major depletion of NK1-expressing neuronal cell bodies and dendrites in lamina I/III (Figure 3C). There was no obvious difference in the amount of NeuN staining between naïve rats and rats treated with SP-SAP or blank-SAP at P48 suggesting that neonatal SP-SAP treatment did not result in global depletion of lamina I/III neurons (Figure 3D, 3E and 3F). There was no difference in immunostaining for GFAP in the dorsal horn between naïve rats and rats treated with SP-SAP at P48 (Figure 4A). In naïve adult rats, expression of PKC gamma is restricted to dorsal horn interneurons in inner lamina II. At P48 there was no difference in immunostaining for PKC gamma between naïve rats and rats treated with SP-SAP at P3 suggesting that PKC gamma expressing neurons were not damaged by neonatal SP-SAP treatment (Figure 4B). In naïve rats CGRP-expressing primary afferents terminate in lamina I and outer lamina II whereas IB4-positive primary afferents terminate in inner lamina II and this was not influenced by neonatal SP-SAP treatment at P3 (Figure 4C,D). In naïve rats, serotonergic fibres terminate within the superficial dorsal horn. There was no qualitative difference in immunostaining for 5-HT in the superficial dorsal horn between P48 naïve rats and P48 rats treated with SP-SAP at P3 (Figure 4E). These findings suggest that NK1-positive lamina I neurons have little or no role in guiding the postnatal elaboration of nociceptive afferents or serotonergic fibres in the superficial dorsal horn and that neonatal treatment with SP-SAP did not cause generalized damage to dorsal horn neurons at maturity.

### Neonatal SP-SAP treatment had no detrimental effects on postnatal weight gain

**Figure 5:**
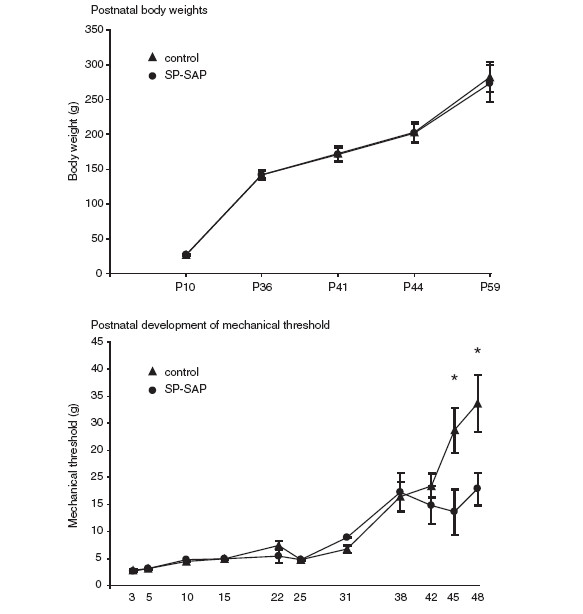
**Neonatal SP-SAP treatment has no effect on postnatal weight gain but results in increased mechanical sensitivity at maturity. A,** Body weight against postnatal age of control rats and rats treated with SP-SAP at P3. **B**, Mechanical threshold of the left hindpaw against postnatal age in control rats and rats treated with SP-SAP at P3. Control group (N = 6; naive and blank-SAP combined), SP-SAP (N = 4). Data show mean ± SEM. * *p* < 0.05.

Body weights of control rats (naïve rats and rats treated with blank-SAP at P3, N = 6) and rats treated with SP-SAP at P3 (N = 4) were measured at different postnatal time points to exclude detrimental effects of neonatal SP-SAP treatment on postnatal growth. Body weights of all animals increased with postnatal age and treatment with SP-SAP had no effect on body weights compared to control rats at all postnatal time points studied (Figure 5A).

### Neonatal treatment with SP-SAP resulted in increased mechanical sensitivity from P45 onwards

Mechanical threshold of the left hindpaw was measured at different postnatal time points in control rats (N = 6, naive and blank –SAP combined) and rats pre-treated with SP-SAP at P3 (N = 4) to investigate whether postnatal ablation of NK1-positive lamina I neurons influenced development of mechanical threshold (Figure 5B). Mechanical threshold of the left hindpaw increased with postnatal age in all animals. Repeated measures ANOVA revealed that mechanical threshold of animals treated with SP-SAP at P3 became less than that of control animals (*F*_1, 8_ = 14.3, *p* < 0.01). Further post-hoc analysis showed a significant difference at P45 (*p* < 0.05) and P48 (*p* < 0.05). Thus, postnatal ablation of NK1-positive lamina I neurons with SP-SAP resulted in increased mechanical sensitivity compared to animals with intact NK1 pathway from P45 onwards.

## Discussion

We show that lamina I NK1-expressing projection neurons are present and functional in rats soon after birth and that noxious stimulation of the periphery results in activation of neurons in the parabrachial nucleus (PB). Ablation of lamina I neurons with SP-SAP at P3 resulted in increased mechanical sensitivity from P45 onwards compared to controls.

Previously, receptor autoradiography with ^125^I] Bolton-Hunter substance P had not demonstrated expression of NK1 receptors in the superficial dorsal horn of newborn rats [26,27]. However we found NK1 staining in superficial dorsal horn neurons from the earliest time point studied (P3). These differences are undoubtedly methodological demonstrating possibly the increased sensitivity and specificity of immunohistochemical methods and the likelihood of non-functional substance P binding sites. c-fos protein expression (a marker for neuronal activation [28,29]) was also seen in neurones projecting to PB and NK1-expressing lamina I neurons following noxious stimulation at the earliest age studied (P3). Given that the majority of projection neurons are NK1-expressing in adult rats it seems highly likely that this projection pathway is intact and functional soon after birth if not earlier. This is supported by the observation that neurons within the lateral PB, which receives a strong input from spinal lamina I neurons [9,10,30], express c-fos two hours after formalin stimulation of the hind paw in P3 rat pups [31]. Lamina I projection neurons terminate throughout the brainstem and our fluorogold injections while aimed at the lateral parabrachial nucleus were more extensive than this in the P3 rats studied and may also have labelled lamina I neurons that project to the periaqueductal grey (PAG) and thalamus. This may explain the higher number of lamina I projection neurons in P3 rats compared to adult rats in this study. However, it seems more likely that the increased length of the adult lumbar spinal cord could explain the reduced density of projection neurones in the adult lumbar segment [32].

We show in this study that activation of lamina I neurons by formalin injection in the hindpaw did not change with postnatal age but activation of PB neurons by formalin injection increased with postnatal age. The difference may be attributed to immature synaptic transmission between lamina I projection neurons and PB neurons, rather than that between nociceptive primary afferents and lamina I projection neurons. It has been shown that expression of synaptophysin, an integral membrane glycoprotein of clear synaptic vesicles, increased between P11 and P30 in the parabrachial nucleus of postnatal rats [33] suggesting that neurotransmission is immature during this period of postnatal development. Previous research suggested that descending controls are predominantly facilitatory at birth and lack a strong descending inhibitory component [18]. Stimulation-produced analgesia from the PAG (which projects upon RVM) develops only three weeks after birth [34]. Behaviourally the low mechanical thresholds and diffuse and exaggerated reflexes seen in neonates may be explained by the predominance of descending facilitation in the early postnatal period [17].

In adult rats ablation of the NK1-expressing lamina I pathway by SP-SAP has no effect on mechanical thresholds at baseline but reduces mechanical sensitivity after neuropathic injury implying that spinal-brainstem loops are only engaged following injury to the periphery [2]. In this study, intrathecal injection of SP-SAP in P3 rats resulted in ablation of lamina I NK1-expressing neurons at maturity and increased mechanical sensitivity without injury to the periphery from P45 onwards. Previous investigations showed that it takes greater than 21 days for lamina I NK1-expressing neurons to become functionally impaired by intrathecal injection of SP-SAP in adult rats [2,35]. In postnatal rats, the RVM exclusively facilitates spinal pain transmission up to P21 and shifts to ‘biphasic facilitation and inhibition’ from P28 onwards [18]. Thus in this study NK-1 expressing lamina I projections were lost at the point in which inhibitory influences were developing in rats treated with SP-SAP. Recent studies looking at the neonatal influence of maternal environment suggest that early experience can have profound effects on complex behavioural responses in the adult [36] through epigenetic regulation of DNA. Epigenetic changes that set levels of inhibition have been reported in RVM following the induction of peripheral pain states [37] and rapid changes in the epigenetic regulator MeCP2 have been reported in lamina I NK1 projection neurons following the establishment of inflammatory pain states [38]. It seems highly likely therefore that neonatal ablation of the lamina I pathway would have profound effects on nociceptive thresholds in the adult through regulation of the excitability of descending pathways by epigenetic modification. Our results also raise a number of questions including at what point is a rat’s central nervous system considered adult and why should mechanical sensitivity be higher in lamina –NK1 ablated rats? A recent review of the literature [39] suggested that the rat brain should be considered mature only after around 60 days although the dorsal horn may mature earlier than this. The increased sensitivity of lesioned animals as they approach adulthood may be a function of the disconnection of ascending and descending pathways that are known to set pain sensitivity. Given the absence of the lamina I pathway informing the brain of peripheral injury, dorsal horn neuronal networks may adopt an intermediate level of excitability as the best option given the reduced flow of nociceptive information to the brain. We suggest that this form of homeostatic plasticity is a response to the reduced activity of the networks within the dorsal horn and brainstem following ablation of the ascending nociceptive pathway.

## Competing interests

The authors declare that they have no competing interests.

## Authors’ contributions

SPH designed the study and the experiments. SHWM contributed to the design of the experiments, performed the experiments and analysed the data. SMG analysed the data. All authors contributed to the writing of the manuscript and approved the final manuscript.

## References

[B1] Mantyh PW Rogers SD Honore P Allen BJ Ghilardi JR Li J Daughters RS Lappi DA Wiley RG Simone DA Inhibition of hyperalgesia by ablation of lamina I spinal neurons expressing the substance P receptor Science 1997 278 275 279 10.1126/science.278.5336.275 9323204

[B2] Nichols ML Allen BJ Rogers SD Ghilardi JR Honore P Luger NM Finke MP Li J Lappi DA Simone DA Mantyh PW Transmission of chronic nociception by spinal neurons expressing the substance P receptor Science 1999 286 1558 1561 10.1126/science.286.5444.1558 10567262

[B3] Suzuki R Morcuende S Webber M Hunt SP Dickenson AH Superficial NK1-expressing neurons control spinal excitability through activation of descending pathways Nat Neurosci 2002 5 1319 1326 10.1038/nn966 12402039

[B4] Todd AJ McGill MM Shehab SA Neurokinin 1 receptor expression by neurons in laminae I, III and IV of the rat spinal dorsal horn that project to the brainstem Eur J Neurosci 2000 12 689 700 10.1046/j.1460-9568.2000.00950.x 10712649

[B5] Torsney C MacDermott AB Disinhibition opens the gate to pathological pain signaling in superficial neurokinin 1 receptor-expressing neurons in rat spinal cord J Neurosci 2006 26 1833 1843 10.1523/JNEUROSCI.4584-05.2006 16467532 PMC6793628

[B6] Todd AJ Puskar Z Spike RC Hughes C Watt C Forrest L Projection neurons in lamina I of rat spinal cord with the neurokinin 1 receptor are selectively innervated by substance p-containing afferents and respond to noxious stimulation J Neurosci 2002 22 4103 4113 12019329 10.1523/JNEUROSCI.22-10-04103.2002 PMC6757649

[B7] Ikeda H Heinke B Ruscheweyh R Sandkuhler J Synaptic plasticity in spinal lamina I projection neurons that mediate hyperalgesia Science 2003 299 1237 1240 10.1126/science.1080659 12595694

[B8] Ikeda H Stark J Fischer H Wagner M Drdla R Jager T Sandkuhler J Synaptic amplifier of inflammatory pain in the spinal dorsal horn Science 2006 312 1659 1662 10.1126/science.1127233 16778058

[B9] Gauriau C Bernard JF Pain pathways and parabrachial circuits in the rat Exp Physiol 2002 87 251 258 10.1113/eph8702357 11856971

[B10] Gauriau C Bernard JF A comparative reappraisal of projections from the superficial laminae of the dorsal horn in the rat: the forebrain J Comp Neurol 2004 468 24 56 10.1002/cne.10873 14648689

[B11] Szucs P Luz LL Lima D Safronov BV Local axon collaterals of lamina I projection neurons in the spinal cord of young rats J Comp Neurol 2010 518 2645 2665 20506469 10.1002/cne.22391

[B12] Hunt SP Pain control: breaking the circuit Trends Pharmacol Sci 2000 21 284 287 10.1016/S0165-6147(00)01496-6 10918627

[B13] Rahman W Suzuki R Webber M Hunt SP Dickenson AH Depletion of endogenous spinal 5-HT attenuates the behavioural hypersensitivity to mechanical and cooling stimuli induced by spinal nerve ligation Pain 2006 123 264 274 10.1016/j.pain.2006.02.033 16644129

[B14] Porreca F Burgess SE Gardell LR Vanderah TW Malan TP Ossipov MH Lappi DA Lai J Inhibition of neuropathic pain by selective ablation of brainstem medullary cells expressing the mu-opioid receptor J Neurosci 2001 21 5281 5288 11438603 10.1523/JNEUROSCI.21-14-05281.2001 PMC6762871

[B15] Khasabov SG Ghilardi JR Mantyh PW Simone DA Spinal neurons that express NK-1 receptors modulate descending controls that project through the dorsolateral funiculus J Neurophysiol 2005 93 998 1006 15456795 10.1152/jn.01160.2003

[B16] Khasabov SG Rogers SD Ghilardi JR Peters CM Mantyh PW Simone DA Spinal neurons that possess the substance P receptor are required for the development of central sensitization J Neurosci 2002 22 9086 9098 12388616 10.1523/JNEUROSCI.22-20-09086.2002 PMC6757691

[B17] Fitzgerald M The development of nociceptive circuits Nat Rev Neurosci 2005 6 507 520 15995722 10.1038/nrn1701

[B18] Hathway GJ Koch S Low L Fitzgerald M The changing balance of brainstem-spinal cord modulation of pain processing over the first weeks of rat postnatal life J Physiol 2009 587 2927 2935 10.1113/jphysiol.2008.168013 19403624 PMC2718251

[B19] Otsuka M Konishi S Release of substance P-like immunoreactivity from isolated spinal cord of newborn rat Nature 1976 264 83 84 10.1038/264083a0 12474

[B20] Otsuka M Konishi S Substance P and excitatory transmitter of primary sensory neurons Cold Spring Harb Symp Quant Biol 1976 40 135 143 10.1101/SQB.1976.040.01.015 7378

[B21] Aita M Seo K Fujiwara N Takagi R Maeda T Postnatal changes in the spatial distributions of substance P and neurokinin-1 receptor in the trigeminal subnucleus caudalis of mice Brain Res Dev Brain Res 2005 155 33 41 10.1016/j.devbrainres.2004.12.005 15763273

[B22] van Oers HJ de Kloet ER Whelan T Levine S Maternal deprivation effect on the infant's neural stress markers is reversed by tactile stimulation and feeding but not by suppressing corticosterone J Neurosci 1998 18 10171 10179 9822770 10.1523/JNEUROSCI.18-23-10171.1998 PMC6793306

[B23] Paxino G Watson C The rat brain in stereotaxic coordinates 1997 London: Academic Press

[B24] Chaplan SR Bach FW Pogrel JW Chung JM Yaksh TL Quantitative assessment of tactile allodynia in the rat paw J Neurosci Methods 1994 53 55 63 10.1016/0165-0270(94)90144-9 7990513

[B25] Torsney C Fitzgerald M Age-dependent effects of peripheral inflammation on the electrophysiological properties of neonatal rat dorsal horn neurons J Neurophysiol 2002 87 1311 1317 11877505 10.1152/jn.00462.2001

[B26] Charlton CG Helke CJ Ontogeny of substance P receptors in rat spinal cord: quantitative changes in receptor number and differential expression in specific loci Brain Res 1986 394 81 91 2428451 10.1016/0165-3806(86)90084-2

[B27] Kar S Quirion R Neuropeptide receptors in developing and adult rat spinal cord: an in vitro quantitative autoradiography study of calcitonin gene-related peptide, neurokinins, mu-opioid, galanin, somatostatin, neurotensin and vasoactive intestinal polypeptide receptors J Comp Neurol 1995 354 253 281 10.1002/cne.903540208 7782502

[B28] Hunt SP Pini A Evan G Induction of c-fos-like protein in spinal cord neurons following sensory stimulation Nature 1987 328 632 634 10.1038/328632a0 3112583

[B29] Doyle CA Hunt SP Substance P receptor (neurokinin-1)-expressing neurons in lamina I of the spinal cord encode for the intensity of noxious stimulation: a c-Fos study in rat Neuroscience 1999 89 17 28 10.1016/S0306-4522(98)00276-0 10051214

[B30] Craig AD Distribution of brainstem projections from spinal lamina I neurons in the cat and the monkey J Comp Neurol 1995 361 225 248 10.1002/cne.903610204 8543660

[B31] McHaffie JG Wang S Walton N Stein BE Redgrave P Covariant maturation of nocifensive oral behaviour and c-fos expression in rat superior colliculus Neuroscience 2002 109 597 607 10.1016/S0306-4522(01)00499-7 11823069

[B32] Walker SM Meredith-Middleton J Lickiss T Moss A Fitzgerald M Primary and secondary hyperalgesia can be differentiated by postnatal age and ERK activation in the spinal dorsal horn of the rat pup Pain 2007 128 157 168 10.1016/j.pain.2006.09.015 17056180

[B33] Lasiter PS Kachele DL Postnatal development of protein P-38 ('synaptophysin') immunoreactivity in pontine and medullary gustatory zones of rat Brain Res Dev Brain Res 1989 48 27 33 10.1016/0165-3806(89)90091-6 2502329

[B34] van Praag H Frenk H The development of stimulation-produced analgesia (SPA) in the rat Brain Res Dev Brain Res 1991 64 71 76 10.1016/0165-3806(91)90210-a 1786649

[B35] Vierck CJ Kline RH Wiley RG Intrathecal substance p-saporin attenuates operant escape from nociceptive thermal stimuli Neuroscience 2003 119 223 232 10.1016/S0306-4522(03)00125-8 12763083

[B36] Caldji C Hellstrom IC Zhang TY Diorio J Meaney MJ Environmental regulation of the neural epigenome FEBS Lett 2011 585 2049 2058 10.1016/j.febslet.2011.03.032 21420958

[B37] Zhang Z Cai YQ Zou F Bie B Pan ZZ Epigenetic suppression of GAD65 expression mediates persistent pain Nat Med 2011 17 1448 1455 10.1038/nm.2442 21983856 PMC3210928

[B38] Geranton SM Morenilla-Palao C Hunt SP A role for transcriptional repressor methyl-CpG-binding protein 2 and plasticity-related gene serum- and glucocorticoid-inducible kinase 1 in the induction of inflammatory pain states J Neurosci 2007 27 6163 6173 10.1523/JNEUROSCI.1306-07.2007 17553988 PMC6672147

[B39] McCutcheon JE Marinelli M Age matters Eur J Neurosci 2009 29 997 1014 10.1111/j.1460-9568.2009.06648.x 19291226 PMC2761206

